# Effect of antiangiogenic-based treatment and systemic inflammatory factors on outcomes in patients with *BRAF* v600-mutated metastatic colorectal cancer: a real-world study in Spain

**DOI:** 10.1186/s12885-020-07758-5

**Published:** 2021-01-14

**Authors:** Nieves Martínez-Lago, Ana Fernández-Montes, Marta Covela, Elena M. Brozos, Juan De la Cámara, José C. Méndez Méndez, Mónica Jorge-Fernández, Antía Cousillas Castiñeiras, Cristina Reboredo, David Arias Ron, María L. Pellón Augusto, Paula González Villarroel, Begoña Graña, Mercedes Salgado Fernández, Alberto Carral Maseda, Francisca Vázquez Rivera, Sonia Candamio Folgar, Margarita Reboredo López, On behalf of the Galician Research Group on Digestive Tumors (GITuD)

**Affiliations:** 1 Medical Oncology Department, University Hospital A Coruña, Xubias de Arriba, 84, 15006 A Coruña, Galicia Spain; 2 Complexo Hospitalario Universitario de Ourense, Ourense, Galicia Spain; 3 Hospital Universitario Lucus Augusti, Lugo, Galicia Spain; 4 Complexo Hospitalario Universitario de Santiago de Compostela, Santiago de Compostela, Galicia Spain; 5 Complexo Hospitalario Universitario de Ferrol, Ferrol, Galicia Spain; 6 Sanatorio Nosa Señora dos Ollos Grandes, Lugo, Galicia Spain; 7 Hospital Universitario Álvaro Cunqueiro, Vigo, Pontevedra Spain; 8 Complexo Hospitalario Universitario de Pontevedra, Pontevedra, Spain

**Keywords:** Antiangiogenic-based chemotherapy, *BRAF* V600E mutations, Metastatic colorectal cancer, Systemic inflammation score

## Abstract

**Background:**

Outcomes are poorer in metastatic colorectal cancer (mCRC) patients with *BRAF* V600E mutations than those without it, but the effect of these mutations on treatment response is unclear. This real-world study assessed the effects of antiangiogenic-based treatment and systemic inflammatory factors on outcomes in patients with *BRAF* V600-mutated mCRC.

**Methods:**

This real-world, multicenter, retrospective, observational study included patients with *BRAF* V600-mutated mCRC treated in eight hospitals in Spain. The primary endpoints were overall survival (OS) and progression-free survival (PFS); overall response rate (ORR) and disease control rate (DCR) were also assessed. The effect of first- and second-line treatment type on OS, PFS, ORR, and DCR were evaluated, plus the impact of systemic inflammatory markers on these outcomes. A systemic inflammation score (SIS) of 1–3 was assigned based on one point each for platelet-lymphocyte ratio (PLR) ≥200, neutrophil-lymphocyte ratio (NLR) ≥3, and serum albumin < 3.6 g/dL.

**Results:**

Of 72 patients, data from 64 were analyzed. After a median of 69.1 months, median OS was 11.9 months and median first-line PFS was 4.4 months. First-line treatment was triplet chemotherapy-antiangiogenic (12.5%), doublet chemotherapy-antiangiogenic (47.2%), doublet chemotherapy-anti-EGFR (11.1%), or doublet chemotherapy (18.1%). Although first-line treatment showed no significant effect on OS, antiangiogenic-based regimens were associated with prolonged median PFS versus non-antiangiogenic regimens. Negative predictors of survival with antiangiogenic-based treatment were NLR, serum albumin, and SIS 1–3, but not PLR. Patients with SIS 1–3 showed significantly prolonged PFS with antiangiogenic-based treatment versus non-antiangiogenic-based treatment, while those with SIS=0 showed no PFS benefit.

**Conclusions:**

Antiangiogenic-based regimens, SIS, NLR, and albumin were predictors of survival in patients with mCRC, while SIS, NLR and serum albumin may predict response to antiangiogenic-based chemotherapy.

**Trial registration:**

GIT-BRAF-2017-01.

## Background

Worldwide, colorectal cancer (CRC) is the third most common cancer in men and the second most common cancer in women, with the age-standardized rate per 100,000 ranging from 31.8 to 51.2 in the countries with the highest incidence [1].

The presence of *BRAF* mutations, which are present in 5–10% of patients with mCRC, is a known adverse prognostic factor, especially in the metastatic setting. Based on retrospective analyses of mCRC patients, those with *BRAF* V600E mutations share common clinical characteristics, such as female gender, older age at diagnosis, and primary tumor location in right-side colon [2]. Molecular classifications of colorectal cancer show that patients with *BRAF* mutations cluster together on the CMS1 subgroup, which is also characterized by high immune activation and infiltration and the co-occurrence of microsatellite instability (MSI) [3].

Systemic inflammation is also known to be prognostic of poor clinical outcomes in patients with CRC [4]. In particular, abnormal acute inflammatory phase proteins (e.g. elevated C-reactive protein [CRP] and decreased serum albumin) and increased counts of neutrophils and platelets relative to lymphocytes have been associated with adverse outcomes [4].

Although we recognize that *BRAF* V600E-mutated CRC is a distinct clinical and biological subgroup, there are no published series on treating these patients in routine clinical practice, and our understanding is based on limited data from clinical trials. Therefore, it is important to further evaluate the prognostic factors that may influence treatment outcomes in patients with *BRAF*-mutated mCRC in a representative cohort. For example, it is unclear how the presence of *BRAF* V600E mutations could predict a poorer response to treatment [5–8].

Controversy remains regarding the choice of first-line therapy in patients with *BRAF*-mutated mCRC. The European Society for Medical Oncology (ESMO) recommends treatment with either doublet or triplet chemotherapy (CT), with the addition of the vascular endothelial growth factor (VEGF) antibody bevacizumab in patients with *BRAF* mutations and epidermal growth factor receptor (EGFR) antibodies in patients without *RAS* mutations [9]. There is also evidence that *BRAF* mutations are predictive of a negative response to EGFR treatment, as indicated in two meta-analyses that showed a lack of benefit from the addition of anti-EGFR treatment to doublet CT in patients with *RAS*-wild type/*BRAF*-mutated mCRC [10, 11].

The future of *BRAF* V600E-mutated CRC treatment is likely to be influenced by the results of ongoing clinical trials of BRAF-targeted treatment strategies. A previous study in patients with *BRAF* V600-mutated non-melanoma cancer showed that monotherapy with the BRAF inhibitor vemurafenib had limited efficacy in CRC patients [12]. Preclinical data has demonstrated the importance of coupling direct BRAF inhibition with EGFR inhibition in order to block signaling pathway feedback loops [13]. Based on these findings, preliminary clinical trial data have demonstrated promising antitumor activity with combined targeted treatment, including vemurafenib coupled with the EGFR inhibitor cetuximab and irinotecan as second- or third-line therapy [14], and ongoing clinical trials are exploring the role of combination BRAF and EGFR inhibition in first-line therapy.

This study, which was initiated before the introduction of BRAF-targeted treatment for mCRC, aimed to elucidate which factors were associated with poorer outcomes in patients with *BRAF* V600-mutated mCRC and whether the ESMO recommendation for doublet CT plus an antiangiogenic agent was associated with improved outcomes by performing a retrospective analysis of clinical outcomes in more than 70 patients with *BRAF* V600-mutated mCRC from eight hospitals in Spain.

## Methods

This was a multicenter, retrospective, observational study in patients with *BRAF* V600-mutated mCRC who treated with the standard of care between 2011 and 2018 at eight hospitals in the Galician Research Group on Digestive Tumors (GITuD) network in the Galicia Autonomous Community, Spain.

The study was conducted in accordance with the Good Clinical Practice Guidelines and the Declaration of Helsinki and obtained the approval of the Ethical Committee of Sanitary Area Santiago-Lugo, Spain (registration code: 2017/453). All patients provided written or oral informed consent before an independent witness of the research team prior to inclusion in the study.

### Eligibility criteria

Patients were eligible for inclusion if they were over 18 years of age and had a confirmed diagnosis of mCRC with the *BRAF* V600 mutation. Exclusion criteria were a history of neoplasm within the previous 5 years (except cervical carcinoma in situ or basal cell carcinoma of the skin); an inability to understand the study procedures or provide informed consent; and incomplete clinical or follow-up data.

### Study endpoints

The primary study endpoints were overall survival (OS) and progression-free survival (PFS). OS was defined as the time from the start of treatment until death by any cause. PFS was defined as the time from treatment start to confirmed radiologic progression or death by any cause.

Secondary endpoints were overall response rate (ORR; defined as the proportion of patients who achieved complete response [CR] or partial response [PR]) and disease control rate (DCR; defined as the proportion of patients who achieved CR, PR, or stable disease [SD] lasting ≥ 6 weeks after the start of treatment).

Exploratory outcomes included the impact of first- and second-line treatment on PFS and OS, and the prognostic impact of clinical factors and systemic inflammation.

Systemic inflammation was assessed using (i) the neutrophil-to-lymphocyte ratio (NLR), with NLR subgroups categorized as ≥ 3 vs < 3; (ii) the platelet-to-lymphocyte ratio (PLR), with PLR subgroups categorized as ≥ 200 vs < 200; and (iii) serum albumin levels (≥ 3.6 g/dL vs < 3.6 g/dL). For each patient, a systemic inflammation score (SIS) was calculated by adding up the number of risk factors, defined as serum albumin < 3.6 g/dL, NLR ≥3 and PLR ≥ 200, with one point assigned to each risk factor.

### Statistical analysis

The primary analysis was conducted using the Kaplan–Meier method to estimate median PFS and OS and 95% confidence intervals (CIs). Differences between survival curves were compared using the log-rank test with a two-sided significance level of 0.05. The chi-squared test or Fisher’s exact test (depending on the sample size) was used to compare clinical and demographic variables. Statistical analyses were carried out using IBM SPPS Statistics V25.0.

## Results

### Patient population

The study included 72 patients who received treatment for mCRC between November 2010 and November 2018. The exploratory efficacy analysis included 64 patients (88.9%); eight patients were excluded from this analysis because they only received palliative care.

Patient baseline characteristics are summarized in Table 1. Median (range) age was 62.4 (30–83) years and 54.2% of patients were female. The median (range) number of metastatic locations was 2 (1–5); 25% of patients had metastases in ≥ 3 locations. Regarding prognostic factors, 27.8% of patients had ECOG PS 2–3, 48.6% had tumors in the right colon, 31.9% had high-grade tumors, 6.9% had deficiency of mismatch repair proteins, and 59.4% had synchronous tumors.

**Table 1:** Patient characteristics and their prognostic impact on overall survival

Characteristics	***N*** = 72	OS (months)	HR (95% CI)	***p***-value^a^
Age, years				
Median	62.4	–	–	–
Range	30–83			
Sex, *n* (%)				
Male	33 (45.8)	9.4	1.317 (0.8–2.2)	0.308
Female	39 (54.2)	12.8		
ECOG PS, *n* (%)				
0–1	52 (72.2)	11.9	0.632 (0.3–1.2)	0.161
2–3	20 (27.8)	7.1		
Tumor location, *n* (%)				
Right-sided	35 (48.6)	10.6	–	0.296
Left-sided	22 (30.6)	11.3		
Rectum	15 (20.8)	13.1		
Histological grade, *n* (%)				
Low grade (G1–2)	37 (51.4)	11.9	0.895 (0.5–1.7)	0.723
High grade (G3)	23 (31.9)	11.1		
Unknown	12 (16.7)			
Mismatch repair proteins, *n* (%)	*n*=33			
Conserved	31 (93.1)	12.8	0.146 (0.1–0.8)	**0.010**
Deficiency	2 (6.9)	4.9		
Tumor presentation, *n* (%)				
Synchronous	50 (69.4)	12.4	0.903 (0.5–1.6)	0.730
Metachronous	22 (30.6)	5.9		
Primary tumor surgery, *n* (%)				
No	25 (34.7)	9.6	1.932 (1.1–3.4)	**0.023**
Yes	47 (65.3)	11.9		
Metastasectomy, *n* (%)				
No	57 (79.2)	9.6	2.307 (1.2–4.6)	**0.011**
Yes	15 (20.8)	19.6		

*CI* Confidence interval, *ECOG PS* Eastern Cooperative Oncology Group performance status, *G* Grade, *HR* Hazard ratio, *OS* Overall survival

^a^Significant values are indicated in bold

### Efficacy

**Fig. 1:**
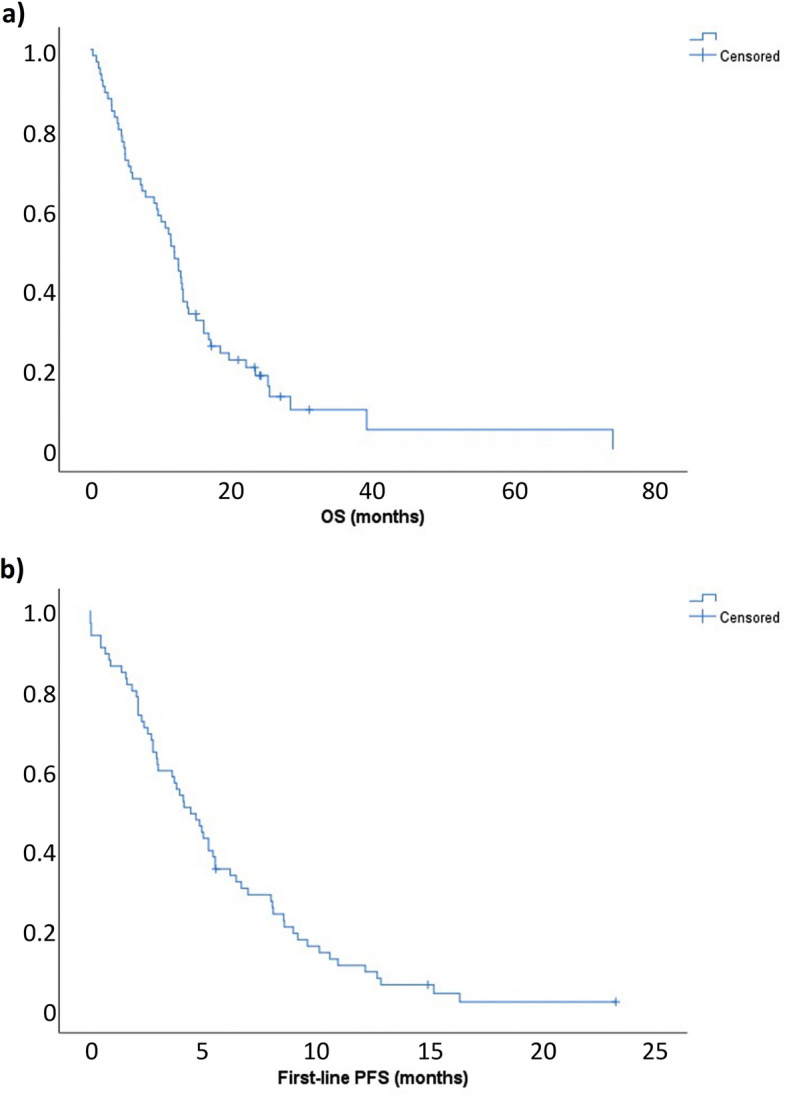
First-line **a** overall survival (OS) and **b** progression-free survival (PFS) in the overall population

In the 64 patients who were evaluated for efficacy, median OS was 11.9 months (95% CI, 9.7–14.0 months) (Fig. 1a) and median first-line PFS was 4.4 months (95% CI, 3.2–5.7 months), after a median follow-up of 69.1 months (Fig. 1b). The best responses achieved were CR in five patients (7.8%), PR in 16 (25.0%), SD in 18 (28.1%), and progressive disease (PD) in 20 (31.3%); response was unevaluable due to clinical decline in five patients (7.8%). When the five unevaluable patients were excluded, 8.5% of patients had a CR, 27.1% had a PR, 30.5% had SD, and 33.9% had PD. The ORR was 35.6% and the DCR was 66.1%.

#### First-line treatment

First-line treatment consisted of triplet CT plus antiangiogenic therapy in nine patients (12.5%), doublet CT plus antiangiogenic therapy in 34 (47.2%), doublet CT plus anti-EGFR therapy in eight (11.1%), and doublet CT in 13 (18.1%).

**Fig. 2:**
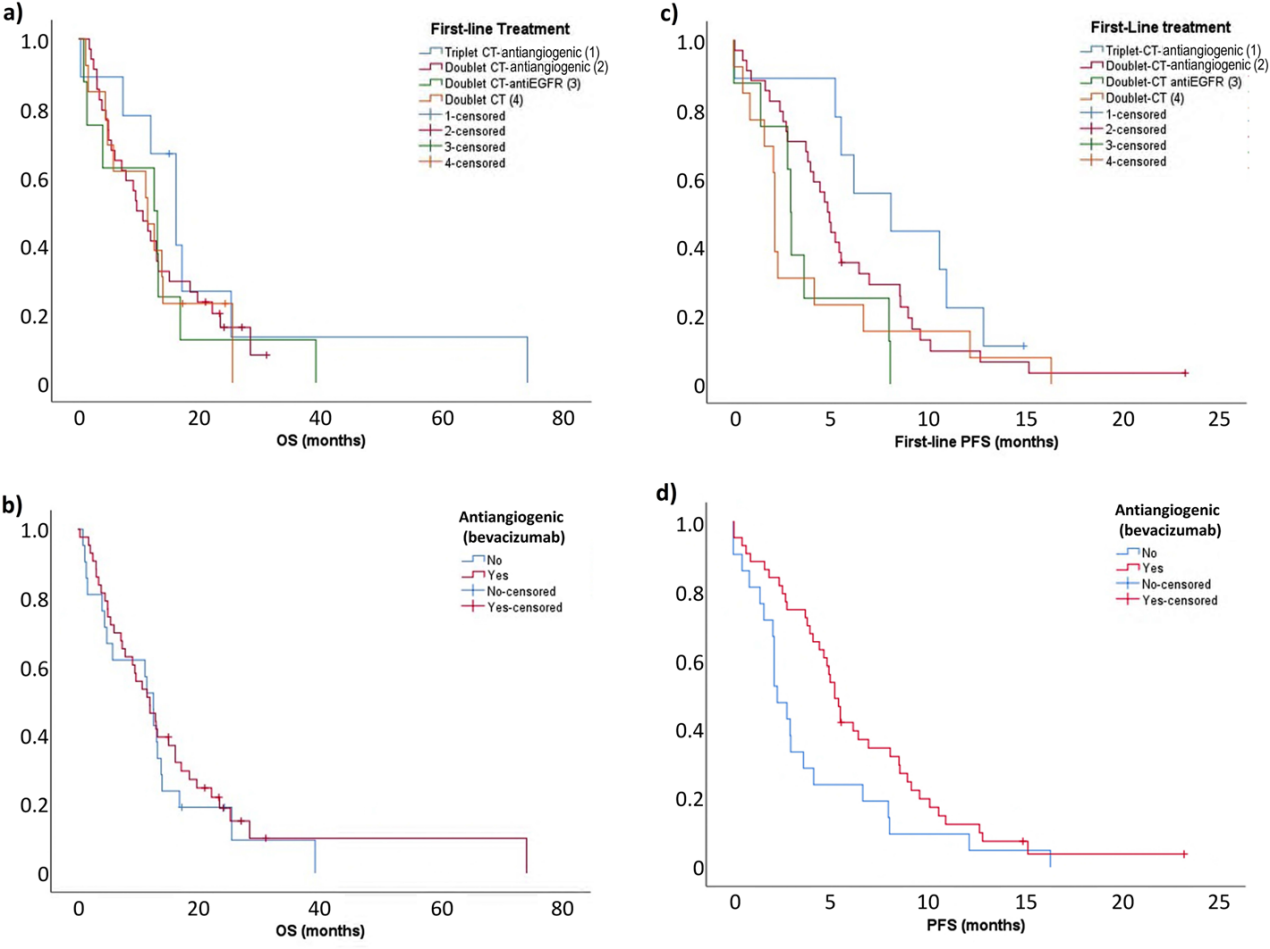
First-line overall survival (OS) **a** across all treatment regimens and **b** comparing first-line regimens with vs without antiangiogenic therapy (bevacizumab), and first-line progression-free survival (PFS) **c** across all treatment regimens and **d** comparing first-line regimens with vs without antiangiogenic therapy (bevacizumab)

There were no statistical differences in OS according to type of treatment, with OS being 16.0 months with triplet CT plus antiangiogenic therapy, 9.6 months with doublet CT plus antiangiogenic therapy, 12.4 months with doublet CT plus anti-EGFR therapy, and 11.3 months with doublet CT (*p* = 0.628) (Fig. 2a). Median OS was 11.8 months in patients who received antiangiogenic-based CT compared with 12.4 months in those who did not (hazard ratio [HR], 1.196; 95% CI, 0.7–2.1; *p* = 0.529) (Fig. 2b).

First-line PFS varied across treatments, being 8.1 months with triplet CT plus antiangiogenic therapy, 4.8 months with doublet CT plus antiangiogenic therapy, 2.9 months with doublet CT plus anti-EGFR therapy, and 2.1 months with doublet CT (*p* = 0.091) (Fig. 2c).

Median first-line PFS was significantly higher in patients treated with antiangiogenic-based CT, being 5.2 months with triplet or doublet CT plus antiangiogenics and 2.3 months with doublet CT with or without anti-EGFR therapy (HR, 0.848; 95% CI, 0.3–0.9; *p* = 0.033) (Fig. 2d). No statistical differences in first-line PFS were found between antiangiogenic-based regimens (triplet CT vs doublet CT HR, 0.543; 95% CI, 0.2–1.2; *p* = 0.123) or between doublet CT with versus without anti-EGFR therapy (HR, 0.989; 95% CI, 0.4–2.4; *p* = 0.929).

First-line ORR based on type of treatment was 75% with triplet CT plus antiangiogenic therapy, 34.4% with doublet CT plus antiangiogenic, 12.5% with doublet CT plus anti-EGFR, and 27.3% with doublet CT (*p* = 0.063; Fisher’s test). ORR was 45.2% with triplet or doublet CT plus antiangiogenic therapy and 21.1% with doublet CT with or without anti-EGFR therapy (*p* = 0.149).

The first-line DCR was 100% with triplet CT plus antiangiogenic therapy, 71.9% with doublet CT plus antiangiogenic, 50.0% with doublet CT plus anti-EGFR, and 36.4% with doublet CT (*p* = 0.015; Fisher’s test). DCR was significantly higher with triplet or doublet CT plus antiangiogenic than with doublet CT with or without anti-EGFR (77.5% vs 42.1%; *p* = 0.017).

#### Second-line treatment

Second-line treatment was required in 61 patients. Second-line treatment consisted of doublet CT plus antiangiogenic therapy in 20 patients (32.8%), doublet CT plus anti-EGFR therapy in three (4.9%), and doublet CT in six (9.8%). This analysis excluded 32 patients; 27 (44.3%) received best supportive care, four (6.6%) received single-agent CT and one (1.6%) enrolled in a clinical trial.

In the 29 patients who were evaluated for second-line efficacy, median PFS was 2.8 months (95% CI, 2.4–3.2 months) and median OS was 5.9 months (95% CI, 3.9–7.2 months). Best responses consisted of PR in five patients (17.2%), SD in eight (27.6%), and PD in 13 (44.8%); response was unevaluable in three patients (10.3%). Excluding the three unevaluable patients, the ORR was 19.2% and the DCR was 50%.

There were no statistical differences in OS according to treatment type, with median second-line OS being 7.1 months with doublet CT plus antiangiogenic therapy, 5.9 months with doublet CT plus anti-EGFR therapy, and 3.4 months with doublet CT (*p* = 0.335). Median second-line OS was 7.1 months in patients who received antiangiogenic-based CT compared with 5.3 months in those who received doublet CT with or without anti-EGFR therapy (HR, 0.532; 95% CI, 0.2–1.2; *p* = 0.140).

Second-line PFS varied numerically across treatments, being 3.5 months with doublet CT plus antiangiogenic therapy, 2.8 months with doublet CT plus anti-EGFR therapy, and 0.1 months with doublet CT (*p* = 0.079). PFS was significantly prolonged in patients treated with second-line antiangiogenic-based CT, being 3.5 months with doublet CT plus antiangiogenic therapy and 2.3 months with doublet CT with or without anti-EGFR therapy (HR, 0.402; 95% CI, 0.2–0.9; *p* = 0.032).

Second-line ORR according treatment type was 26.3% with doublet CT plus antiangiogenic therapy, 0% with doublet CT with or without anti-EGFR therapy (*p* = 0.190). Second-line DCR according to treatment type was 63.2% with doublet CT plus antiangiogenic therapy, 0% with doublet CT plus anti-EGFR therapy, and 25% with doublet CT (*p* = 0.096). The second-line ORR and DCR were significantly higher among patients treated with antiangiogenic-based CT versus doublet CT with or without anti-EGFR therapy (26.3% vs 0%; *p* = 0.153 and 63.2% vs 14.3%; *p* = 0.020, respectively).

### Prognostic factors

Of the patient characteristics that were analyzed as potential prognostic factors, the characteristics that were associated with significantly improved OS were conserved mismatch repair (*p* = 0.010), primary tumor resection (*p* = 0.023), and metastasis resection (*p* = 0.011; Table 1).

#### Systemic inflammation score

Regarding the SIS parameters, 50.7% of patients had NLR ≥ 3, 43.7% had PLR ≥ 200 and 19.4% had serum albumin < 3.6 g/dL (Table 2). NLR ≥ 3 was positively associated with PLR ≥ 200 (*p* < 0.001), but neither NLR (*p* = 0.543) nor PLR (*p* = 0.375) was associated with low serum albumin levels.

**Table 2:** Systemic inflammation parameters and their prognostic impact on overall survival

Parameter	***n*** (%)	Median OS, months	HR (95% CI)	***p***-value^a^
NLR				
< 3	35 (49.3%)	7.8	1.934 (1.1–3.3)	**0.014**
≥ 3	36 (50.7%)	13.7		
PLR				
< 200	40 (56.3%)	5.4	1.567 (0.9–2.7)	0.096
≥ 200	31 (43.7%)	13.0		
Serum albumin levels				
< 3.6 g/dL	14 (19.4%)	4.9	2.142 (1.1–4.5)	**0.040**
≥ 3.6 g/dL	47 (65.3%)	12.4		
Unknown	11 (15.3%)	–	–	–
SIS				
0	25 (35.2%)	16.0	–	**0.004**
1	18 (25.4%)	7.8		
2	21 (29.6%)	5.7		
3	7 (9.9)	4.0		
SIS groups				
0	25 (35.2%)	16.0	0.475 (0.3–0.8)	**0.008**
1–3	46 (64.8%)	7.3		

*CI* Confidence interval, *HR* Hazard ratio, *NLR* Neutrophil-to-lymphocyte ratio, *PLR* Platelet-to-lymphocyte ratio, *SIS* Systemic inflammation score

^a^Significant values are indicated in bold

Both increased NLR (*p* = 0.014) and reduced serum albumin levels (*p* = 0.040) had a significant negative prognostic impact on OS. Increased PLR showed a non-significant trend towards worse survival (*p* = 0.096; Table 2).

**Fig. 3:**
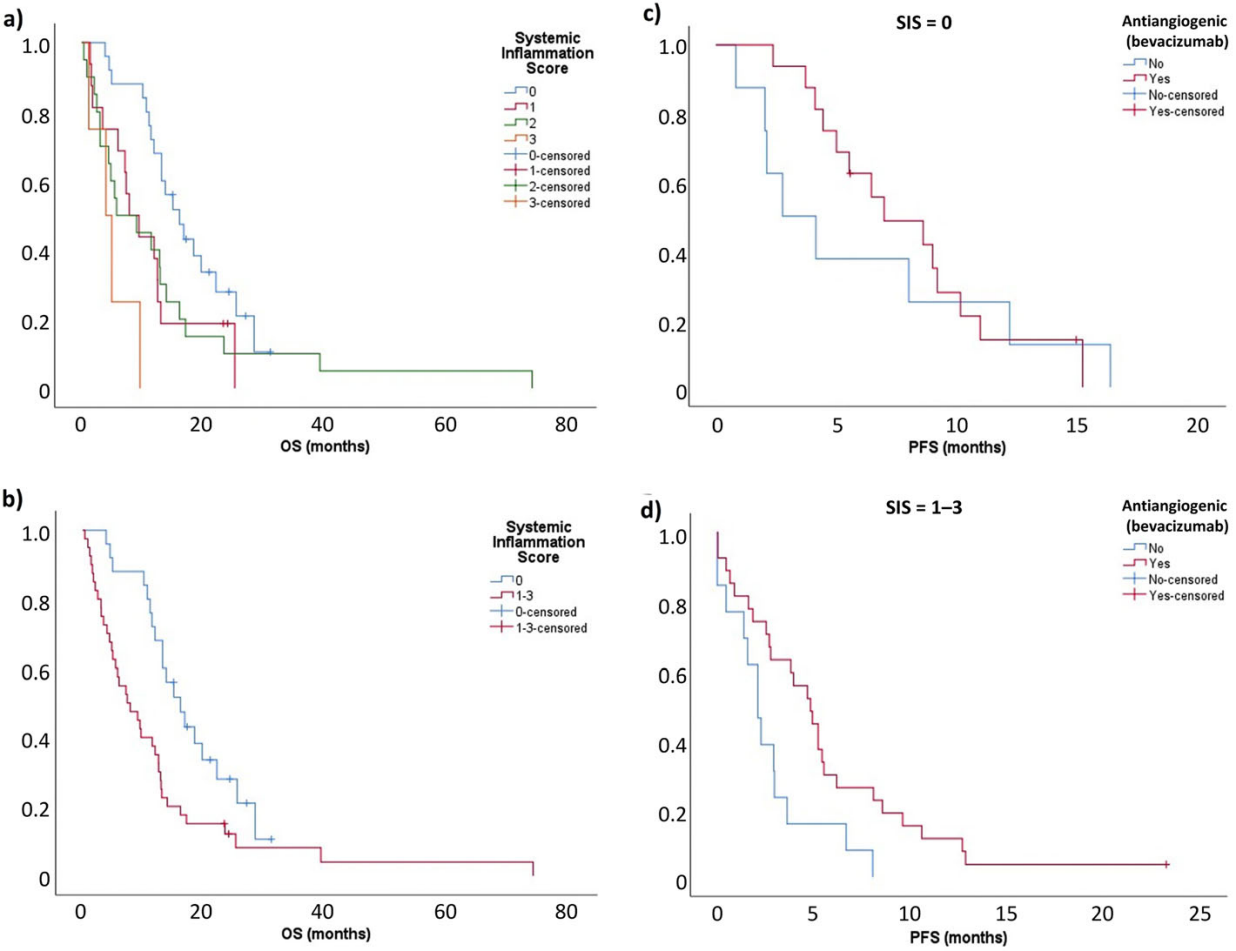
Overall survival (OS) according to systemic inflammation scores (SIS) of **a** 0, 1, 2 or 3 and **b** 1–3 vs 0, and progression-free survival (PFS) with vs without antiangiogenic therapy (bevacizumab) in patients with SIS of **c** 0 and **d** 1–3

The SIS score was predictive of survival; median OS was 16.0 months in patients with no factors, 7.8 months with 1 factor, 5.7 months with 2 factors, and 4.0 months with 3 factors (*p* = 0.004) (Fig. 3a). Patients with an SIS score of 0 also had a significantly longer median OS than those with an SIS score of 1–3 (16.0 vs 7.3 months; HR, 0.475, 95% CI, 0.3–0.8; *p* = 0.008) (Fig. 3b).

There was a significant benefit with antiangiogenic-based CT in patients with an SIS score of 1–3, but not in those with a score of 0. In patients with an SIS score of 1–3, median PFS was 4.8 months for antiangiogenic-based regimens versus 2.1 months for non-antiangiogenic-based CT (HR, 2.340; 95% CI, 1.1–4.8; *p* = 0.016) (Fig. 3c). Conversely, in patients with SIS score 0, median PFS was 7.0 versus 2.8 months, respectively (HR, 1.266; 95% CI, 0.5–3.2; *p* = 0.616) (Fig. 3d).

## Discussion

This retrospective study of 72 patients with *BRAF* V600-mutated mCRC is the largest published series to represent the wide spectrum of patients that are seen in routine clinical practice. Overall, the relatively short median OS (11.9 months) and first-line median PFS (4.4 months), combined with the fact that eight patients could not receive any active treatment, confirms previous observations that *BRAF* mutations confer a worse prognosis and poorer response to treatment than those with wild-type *BRAF* tumors when associated with advanced disease at diagnosis. For example, in a study of mCRC patients who were treated with first-line doublet CT plus anti-EGFR therapy (panitumumab), median OS was 9.2–10.4 months and a median PFS was 5.4–6.0 months in patients with *BRAF*-mutated tumors compared with 15.1–40.0 months and 7.8–12.6 months, respectively, in those with wild-type *BRAF* mCRC [15].

Interestingly, although left-side tumors were present in almost 50% of the patients in our study, only 6.9% had alterations in mismatch repair genes. A Swedish study previously suggested that the percentage of MSI alteration in early stage *BRAF*-mutated CRC might reach 50%, but in this case the presence of MSI as a positive prognostic factor overcame the negative prognosis conferred by *BRAF* mutation [16], meaning that most patients with left-sided BRAF-mutated tumors and MSI may not develop metastases. The Swedish study also found that rectal *BRAF*-mutated tumors had an adverse prognosis and were not associated with MSI [16].

The ESMO clinical guidelines recommend first-line treatment with a combination of doublet or triplet CT plus an antiangiogenic therapy in patients with *BRAF*-mutated mCRC to increase the first-line response rate and PFS [9]. Because the start of our real-world study predates the publication of this ESMO recommendation, our study aimed to show that this approach led to better outcomes in patients with *BRAF*-mutated mCRC. In fact, almost 60% of patients received first-line treatment with doublet or triplet CT plus antiangiogenic therapy (i.e. bevacizumab), and antiangiogenic-based treatment was associated with notably longer PFS and greater DCR than non-antiangiogenic-based treatment. Although there was no difference in OS when stratified according to presence or absence of antiangiogenic therapy, the small number of patients in each treatment group means that this analysis may have lacked the statistical power to detect a difference in OS.

The potential benefit of adding antiangiogenic therapy in the general CRC patient population is unclear. For example, a phase III, multicenter, randomized trial found no clinical benefit of adding bevacizumab to FOLFOX4 or FOLFIRI in first-line treatment of mCRC patients [17], while a meta-analysis showed that bevacizumab significantly reduced mortality risk in primary tumor-resected mCRC patients compared with patients without primary tumor resection [18]. In another meta-analysis, this one in patients with mutated *BRAF* V600E who received second-line treatment with FOLFIRI ± an antiangiogenic within the TRIBE, TRIBE-2, VELOUR and RAISE studies, anti-angiogenics were found to have a significant advantage over placebo in terms of OS (HR, 0.50, 95% CI, 0.29–0.85; *p* = 0.01) [19], suggesting that patients with *BRAF*-mutated tumors might benefit specially from antiangiogenic therapy. The reason why our study found a potential beneficial effect of antiangiogenic therapy might reside in the particular importance of inflammation in this molecular subgroup.

Due to the timeframe of data accrual, patients in our study received BRAF inhibitors, such as encorafenib. Single-agent BRAF inhibitor treatment has demonstrated limited effectiveness against *BRAF* V600-mutated mCRC [20], because mCRC tumors harboring *BRAF* mutations rapidly develop adaptive resistance to RAS [21]. Combining a BRAF inhibitor with EGFR or MEK inhibitors may result in improved outcomes [21]. Indeed, in the BEACON trial in patients with *BRAF* V600-mutated mCRC, second-line treatment with a BRAF inhibitor (encorafenib), an anti-EGFR antibody (cetuximab), and a MEK inhibitor (binimetinib) resulted in significantly prolonged median OS compared with control (9.0 vs 5.4 months; *p* < 0.001) [22]. Therefore, although first-line treatment with CT plus anti-EGFR showed no survival benefit, the combination of anti-EGFR therapy plus BRAF inhibitors may be a new standard for second-line treatment of patients with *BRAF*-mutated mCRC.

In our study, we evaluated the prognostic and predictive value of a set systemic inflammation markers that have been previously described elsewhere [4], and used them to create a prognostic score tool. PLR and NLR have been studied as prognostic factors in most tumor types [23], and hypoalbuminemia has been identified as a prognostic indicator in CRC [24]. The majority of patients (64.8%) with *BRAF*-mutated CRC in our study displayed at least one inflammation marker, confirming the significance of inflammation in this subgroup. Both increased NLR and reduced serum albumin had a significant negative prognostic impact on OS in our study, while PLR showed a non-significant trend. The SIS was also correlated with OS, with median OS being highest in patients with a score of 0 and lowest in those with a score of 3. There was a treatment benefit of antiangiogenic therapy in patients with any systemic inflammation (SIS 1–3) but not in those with a score of 0, although PFS was numerically prolonged in this subgroup. This most likely reflects a lack of statistical power, rather than there being no benefit in patients with no inflammatory factors. In a study in patients with mCRC receiving bevacizumab, PFS was longer in patients with low NLR (< 3.44) than those with high NLR (≥ 3.44), and in patients who had received anti-EGFR therapy, PFS was longer in patients with low PLR (< 160.66) versus high PLR (≥ 160.66) [23]. Although this study did not show a significant impact of PLR or NLR on PFS, this suggests that inflammatory markers may predict benefit of bevacizumab in CRC. While our study found hypoalbuminemia to be a negative prognostic indicator on its own, previous studies, including that of McMillan and colleagues, found only elevated CRP levels, not hypoalbuminemia, to be negative predictors of survival [25]. However, hypoalbuminemia combined with CRP is part of the modified Glasgow Prognostic Scale (mGPS) [25]. The risk of death has been found to be significantly increased in patients with a mGPS score of 2 versus 0 (*p* = 0.017) [26]. Although our study found that none of the SIS parameters were predictive of response to anti-EGFR therapy, this may have been an effect of the small number of patients treated with anti-EGFR therapy.

The limitations of this study include the retrospective nature of its study design, the lack of a control arm, and the relatively small numbers of patients in some treatment categories.

## Conclusions

This study confirms that the *BRAF* V600E mutation is a prognostic indicator of poor outcomes in patients with mCRC. Antiangiogenic-based regimens and systemic inflammatory markers, including elevated PLR and NLR and decreased serum albumin, are prognostic in this population, while SIS, elevated NLR, and decreased serum albumin may also predict response to first-line antiangiogenic-based treatment.

## Data Availability

Data are available upon reasonable request.

## Abbreviations

CIs: Confidence intervals
CR: Complete response
CRC: Colorectal cancer
CRP: C-reactive protein
CT: Chemotherapy
DCR: Disease control rate
EGFR: Epidermal growth factor receptor
ESMO: European Society for Medical Oncology
GITuD: Galician Research Group on Digestive Tumors
HR: Hazard ratio
mCRC: Metastatic colorectal cancer
mGPS: Modified Glasgow Prognostic Scale
MSI: Microsatellite instability
NRL: Neutrophil-lymphocyte ratio
ORR: Overall response rate
OS: Overall survival
PD: Progressive disease
PLR: Platelet-lymphocyte ratio
PFS: Progression-free survival
PR: Partial response
SD: Stable disease
SIS: Systemic inflammation score
VEGF: Vascular endothelial growth factor
